# *Dmrt1* is required for primary male sexual differentiation in Chinese soft-shelled turtle *Pelodiscus sinensis*

**DOI:** 10.1038/s41598-017-04938-5

**Published:** 2017-06-30

**Authors:** Wei Sun, Han Cai, Gloria Zhang, Haiyan Zhang, Haisheng Bao, Li Wang, Jian Ye, Guoying Qian, Chutian Ge

**Affiliations:** 1 College of Biological and Environmental Sciences, Zhejiang Wanli University, Ningbo, 315100 China; 2 Trinity School of Arts and Sciences, Duke University, Durham, NC 27708 USA; 3 College of Fisheries and Life Sciences, Shanghai Ocean University, Shanghai, 201306 China; 4 HangZhou Aquacultural Technique Extending Centre, Hangzhou, 310001 China

## Abstract

In vertebrates, the primary sex-determining signals that initiate sexual development are remarkably diverse, ranging from complete genetic to environmental cues. However, no sex determination-related genes have been functionally identified in reptiles. Here, we characterized a conserved DM domain gene, *Dmrt1,* in Chinese soft-shelled turtle *Pelodiscus sinensis* (*P*. *sinensis*), which exhibits ZZ/ZW sex chromosomes. *Dmrt1* exhibited early male-specific embryonic expression, preceding the onset of gonadal sex differentiation. The expression of *Dmrt1* was induced in ZW embryonic gonads that were masculinized by aromatase inhibitor treatment. *Dmrt1* knockdown in ZZ embryos by RNA interference resulted in male to female sex reversal, characterized by obvious feminization of gonads, significant down-regulation of testicular markers *Amh* and *Sox9*, and remarkable up-regulation of ovarian regulators, *Cyp19a1* and *Foxl2*. Conversely, ectopic expression of *Dmrt1* led to largely masculinized genetic females, production of *Amh* and *Sox9*, and a decline in *Cyp19a1* and *Foxl2*. These findings demonstrate that *Dmrt1* is both necessary and sufficient to initiate testicular development, thereby acting as an upstream regulator of the male pathway in *P*. *sinensis*.

## Introduction

Chinese soft-shelled turtle *Pelodiscus sinensis* (*P*. *sinensis*), belonging to the Reptilia, Testudines, turtle family, is an important aquaculture species in southern China. This species exhibits a sex-dependent dimorphic growth pattern. Compared with females, male individuals are characterized by larger body size, faster growth, thicker and wider calipash, and less fat^1^. Recently, researchers have been exploring the sex control approach to give rise to all male turtle offspring. The sex determination mode of *P*. *sinensis* is generally categorized as genetic sex determination (GSD), as evidenced by the existence of heteromorphic ZZ/ZW micro-sex chromosomes^2,3^. However, little is known about the genetic components involved in primary sex determination and gonadal differentiation in *P*. *sinensis*.

In vertebrates, the primary sex-determination signals that initiate gonadal differentiation vary remarkably, ranging from complete genetic to environmental cues (for example, temperature-dependent sex determination, TSD). Most studies of primary genetic signals have been focused on the identification of sex-determining genes and understanding its downstream molecular genetic network. For example, *Sry*, the first discovered SD gene, has been proven to function as the master initiator for testicular development by activating its direct target *Sox9* transcription during a crucial period in eutherian mammals^4–8^. Besides *Sry*, several other SD genes or candidates have been identified sequentially, such as *Dmrt1* in chicken^9,10^, *Dmw* in frog^11,12^, and *Dmy*^13,14^, *Amhy*^15^, *Amhr2*^16^, *SdY*^17^, *Gsdf* ^18^, *Sox3*^19^ and *Gdf6Y* ^20^ in fish^21^. However, functional characterization of SD-related genes has not yet been reported in reptilian species.

In spite of the large diversity in primary genetic sex determination signals, the *Dmrt1* gene that codes for a transcription factor with a DNA-binding motif (DM domain), seems to be an evolutionary conserved factor that participates in primary sex determination and postnatal testicular differentiation^22^. *Dmrt1* exhibits a sexually dimorphic expression pattern and knock out/down of this gene causes male to female sex reversal in many taxa^9,13,23,24^. Indeed, in some non-mammalian vertebrates lacking *Sry*, *Dmrt1* or its homologous gene acts as a regulator that is analogous to *Sry* in primary sex determination and sexual differentiation. For example, in chicken^9,10^ and medaka fish *Oryzias latipes*^13,14^, *Dmrt1* and *Dmy*, located on Z and Y sex chromosome respectively, have been demonstrated to be necessary and sufficient to initiate male development. In the frog *Xenopus laevis*, a duplicated and truncated *Dmrt1* gene on the female-specific W chromosome, *Dmw*, is required for female sex determination^11^. Interestingly, the *Dmw* gene triggers ovarian development via blocking the ability of the autosomal *Dmrt1* gene to determine testis fate^12^. In reptiles, DM domain genes have mainly been cloned and characterized in several species exhibiting TSD. Male-specific up-regulation of *Dmrt1* in early developing gonads, prior to the onset of gonadal differentiation, has been observed in the red-eared slider turtle^25,26^, suggesting a possible upstream role of *Dmrt1* in determining the fate of the bipotential gonad in turtles. However, a complete functional study of *Dmrt1* in turtles has not yet been performed, which is largely due to lack of an effective genetic manipulation approach.

Herein lentiviral vector-mediated RNA interference and over expression system was introduced into embryos of *P*. *sinensis* to manipulate the ectopic expression of *Dmrt1*, elucidating the functional role of *Dmrt1* on primary sex determination in *P*. *sinensis*. The male-specific expression of *Dmrt1* was identified in the nucleus of pre-Sertoli cells during the sex determination period, which preceded the initiation of gonadal sex differentiation. The expression of *Dmrt1* was induced in genetic female embryonic gonads that were masculinized by aromatase inhibitor treatment. Loss- and gain-of-function analyses provided the solid evidence that *Dmrt1* is necessary for primary male sexual differentiation, and its ectopic expression is sufficient to trigger testicular development, thereby functioning as an upstream regulator in male sexual differentiation in *P*. *sinensis*.

## Results

### Characterization of *P*. *sinensis Dmrt1* gene

The full-length coding sequence of *P*. *sinensis Dmrt1* was obtained by RT-PCR and 5′ and 3′ RACE. The complete cDNA sequence of *P*. *sinensis Dmrt1* was 2,409 base pairs (bp) (accession number KY964413), with a 230 bp 5′ untranslated region (UTR), a 1072 bp 3′ UTR, and an open reading frame (ORF) of 1,107 bp, which encodes a protein of 368 amino acid (aa) (Supplementary Fig. 1a). The DM domain that is present in mice and chicken *Dmrt1* was also highly conserved in *P*. *sinensis Dmrt1*. The deduced amino acid sequence of *P*. *sinensis Dmrt1* shared 98.4%, 80.7%, 75.7%, 75.3%, 71.2%, 53.9% and 47.3% identity with that of the red-eared slider turtle (*Trachemys scripta*), chicken (*Gallus gallus*), human (*Homo sapiens*), mice (*Mus musculus*), frog (*Xenopus laevis*), zebrafish (*Danio rerio*), Medaka (*Oryzias latipes*), respectively (Supplementary Fig. 1b). The phylogenetic tree showed that *P*. *sinensis Dmrt1* was evolutionarily most closely related to the red-eared slider turtle, followed by chicken, human and mice, and distantly related to fish (Supplementary Fig. 1c).

**Figure 1:**
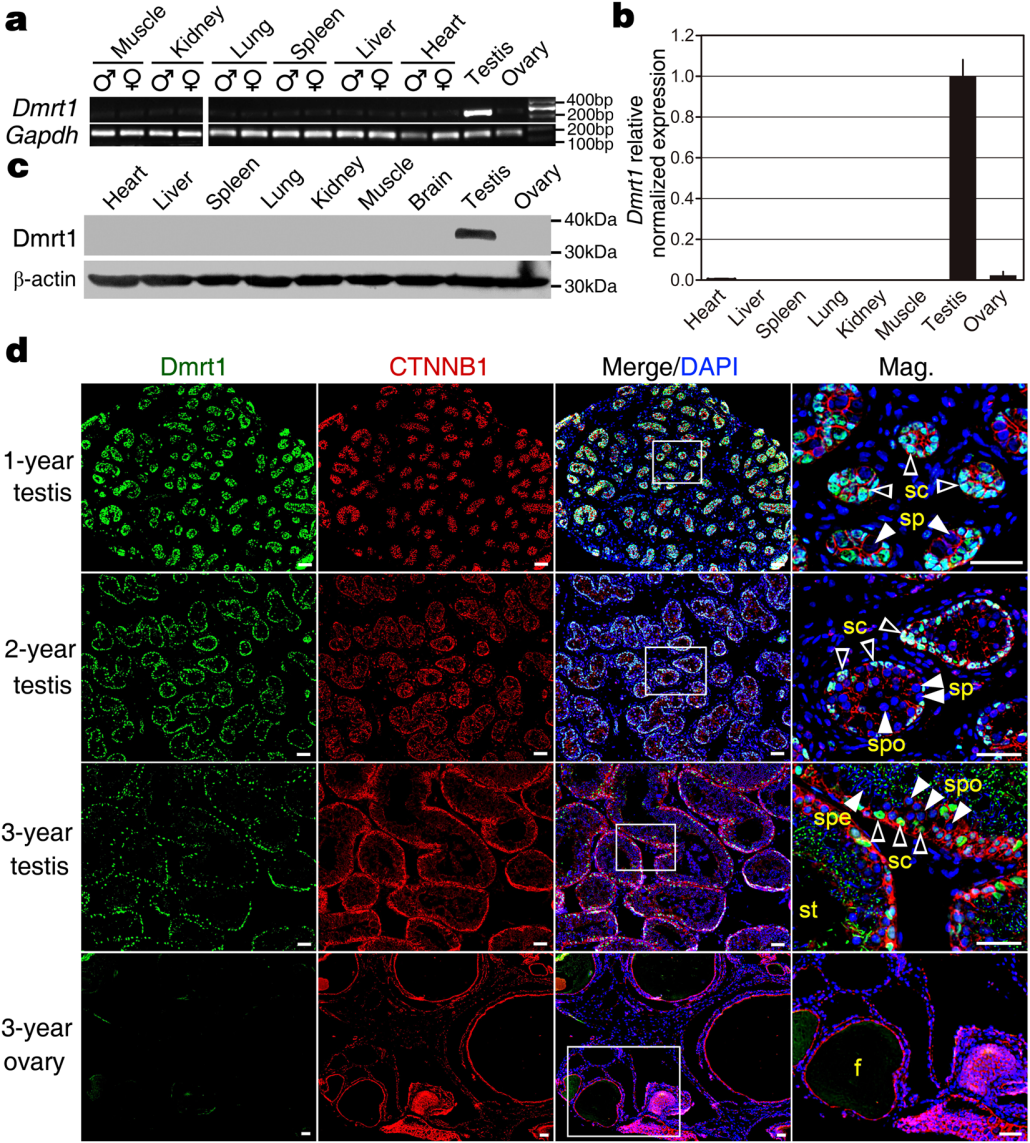
The testis-specific expression of *Dmrt1* in *Pelodiscus sinensis*. (**a**,**b**) The expression of *Dmrt1* mRNA in different tissues were analyzed by RT-PCR and qRT-PCR, respectively. Data are shown as means ± S.D. N ≥ 3. Full-length gels were presented in Supplementary Fig. 2a. (**c**) The expression of Dmrt1 protein in different tissues was examined by western blot. Full-length blots were presented in Supplementary Fig. 2b. (**d**) Immunofluorescence of Dmrt1 (green) were performed in 1-, 2-, 3-year testis, showing robust expression in the nuclei of Sertoli cells. Almost no cells were Dmrt1 postive in 3-year ovary. CTNNB1 immunofluorescence (red) were performed to better display gonadal structure morphology. Nuclei were stained with DAPI (blue). sc, Sertoli cell; sp, spermatogonium; spo, spermatocyte; spe, sperm; st, seminiferous tubule; (**f**) follicle. Scale bars are 50 μm.

In this study, the mRNA and protein expressions of *Dmrt1* were first examined in different tissues of adult turtle. Using RT-PCR, qRT-PCR and Western blot analyses (Fig. 1a,b,c and Supplementary Fig. 2a,b), we determined that *Dmrt1* was abundantly expressed in the testis, but was not detected in the ovary, heart, liver, spleen, lung, kidney, and muscle. In addition, we examined the cellular localization of Dmrt1 protein in 1-year, 2-year and 3-year old testes of *P*. *sinensis* by immunofluorescence. Dmrt1 protein was localized in the nucleus of Sertoli cells surrounding the spermatogonia in testes but was not detected in germ cells of different developmental stages. Meanwhile, we examined the Dmrt1 localization in 3-year old ovaries. As expected, no Dmrt1 fluorescence signal was observed in ovaries of this stage (Fig. 1d).

### Dimorphic expression pattern of *Dmrt1* in early embryonic gonads

**Figure 2:**
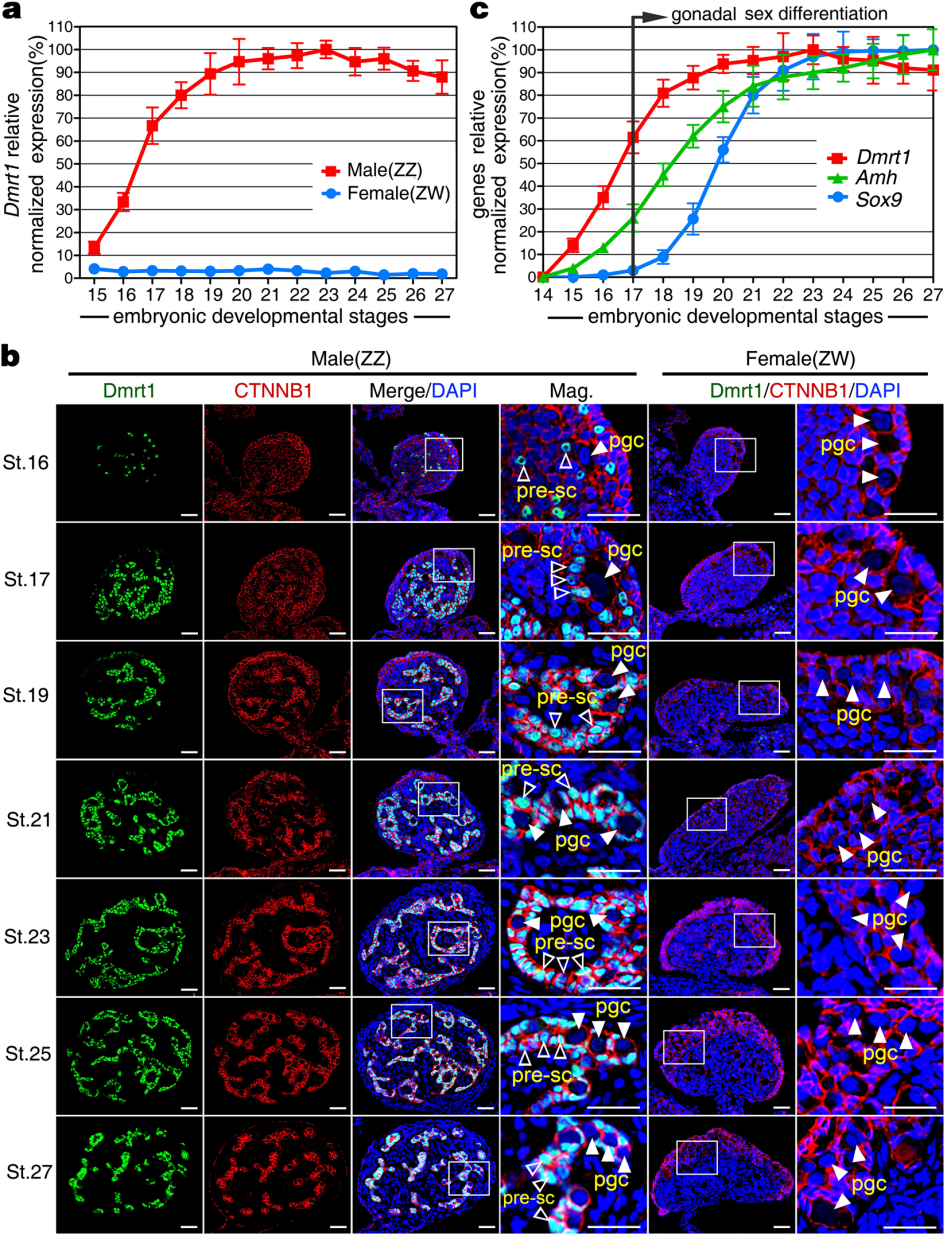
Dimorphic expression pattern of *Dmrt1* in early embryonic gonads. (**a**) qRT-PCR showed the expression of *Dmrt1* mRNA in embryonic gonads of both sexes. *Dmrt1* was initially expressed in male gonads at stage 15, dramatically increased at stage 16 and then maintained at a high level from stage 20. However, extremely low level of *Dmrt1* expression was detected in female gonads throughout embryogenesis. Data are shown as means ± S.D. N ≥ 3. (**b**) Localization of Dmrt1 protein in developing embryonic gonads of both sexes was performed via double immunofluorescence of Dmrt1 and CTNNB1. Dmrt1 protein was mainly localized in the nuclei of precursor sertoli cell surrounding primordial germ cells in male gonads. No Dmrt1 protein signals was detectable in female gonads throughout all time points. pre-sc, precursor sertoli cell; pgc, primordial germ cells. Scale bars are 50 μm. (**c**) Expression patterns of *Dmrt1*, *Amh* and *Sox9* mRNA during embryogenesis were examined by qRT-PCR. Onset of *Dmrt1* expression in male gonads occurred before gonadal sex differentiation, preceding *Amh* and *Sox9*. Data are shown as means ± S.D. N ≥ 3.

To find out whether *Dmrt1* gene is involved in male sexual differentiation in *P*. *sinensis*, we first analyzed the expression pattern of *Dmrt1* mRNA in early embryonic gonads of different developmental stages by qRT-PCR. Here, each embryo’s sex was identified by karyotype analysis and qPCR of 18S rRNA gene. *Dmrt1* transcript was detected in male gonads throughout the period of gonadal sex differentiation from as early as stage 15, and increased dramatically to a peak of stage 23. In contrast, female gonads exhibited extremely low expression of *Dmrt1* throughout embryogenesis (Fig. 2a).

We then proceeded to examine the expression and cellular localization of Dmrt1 protein in early embryonic gonads through immunofluorescence. Here, β-catenin (CTNNB1) immunofluorescence was performed to better display the structure of turtle embryonic gonads^27^. The embryonic gonads were morphologically undifferentiated and appeared identical between the sexes at stage 16 and 17 (prior to the onset of sexual differentiation). At this time, Dmrt1 protein was already strongly expressed throughout the medulla of male gonads, whereas it was not detected in females (Fig. 2b). At stage 19, formation of the primitive seminiferous cord occurred only in medullar region of male gonads. Dmrt1 protein was abundantly expressed and mainly localized in the nuclei of precursor Sertoli cells surrounding the primordial germ cells in male gonads. From stage 21 to 27, Dmrt1 protein continued to be widespread in the medulla of male gonads, and was maintained at a high expression level. However, Dmrt1 protein signal was not detectable in female gonads throughout embryogenesis (Fig. 2b).

In addition, we analyzed the chronological expression of *Dmrt1*, *Amh*, and *Sox9*, which are key factors involved in male development. qRT-PCR analyses revealed that initiation of *Dmrt1* and *Amh* expression in male gonads occurred before gonadal sex differentiation, and the onset of *Dmrt1* at stage 15 preceded that of *Amh* at stage 16. *Sox9* transcript was first detectable at stage 18, when gonadal sex differentiation had begun (Fig. 2c).

### Up-regulation of *Dmrt1* in ZW embryonic gonads during female to male sex reversal

Previous studies in our laboratory have demonstrated that treatment of exogenous estradiol-17β (E_2_) at periods of primary sex determination were able to induce ZZ embryos to develop towards the female phenotype (male to female sex reversal), whereas aromatase inhibitor (AI) was capable of masculinizing ZW embryos (female to male sex reversal)^28^. Here, genotype of all E_2_/AI-treated embryos was determined by karyotype analysis and qPCR of 18S rRNA gene. The masculinized ZW embryos exhibited male-like morphology with dense medulla and degenerated cortex (Supplementary Fig. 3a). A significant increase in *Amh* and *Sox9*, and down-regulation of *Cyp19a1* and *Foxl2* were detected in masculinized ZW embryos by qRT-PCR (Supplementary Fig. 3b). Meanwhile, a thick outer cortex and degenerated medulla, as well as decrease of *Amh* and *Sox9* and up-regulation of *Cyp19a1* and *Foxl2*, were observed in feminized ZZ embryos (Supplementary Fig. 3).

**Figure 3:**
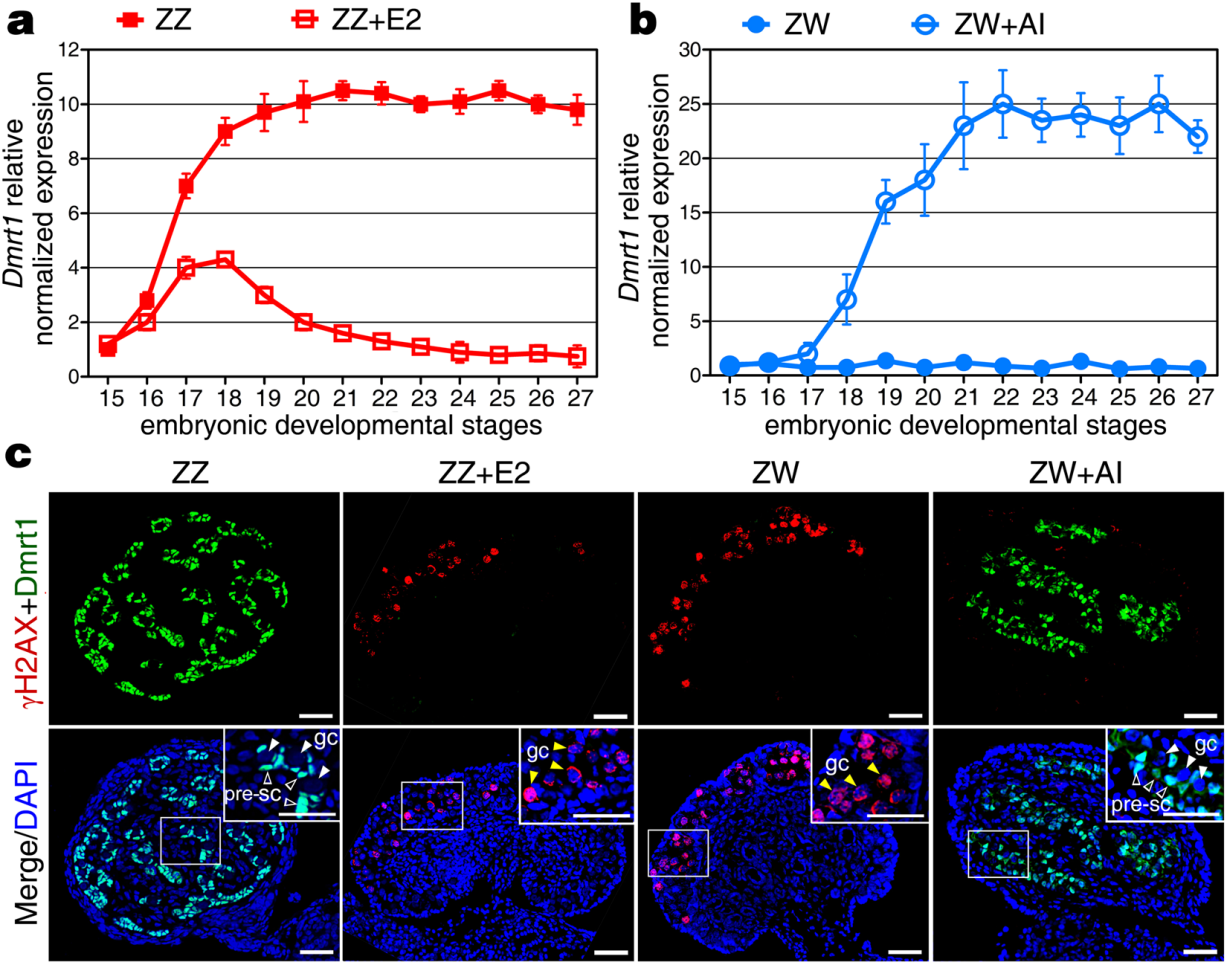
Upregulation of *Dmrt1* in ZW embryonic gonads during female to male sex reversal. (**a**) qRT-PCR analysis showed that *Dmrt1* mRNA expression exhibited a dramatic down-regulation in E_2_-treated ZZ gonads from stage 17 to 27, compared to the control ZZ gonads. Data are shown as means ± S.D. N ≥ 3. (**b**) In AI-treated ZW gonads, *Dmrt1* expression was induced from stage 18, reached the peak at stage 22, and maintained at high level since that time. Data are shown as means ± S.D. N ≥ 3. (**c**) Double immunofluorescence of Dmrt1 and γH2AX was performed in sections of the control ZZ gonads, E_2_-treated ZZ gonads, the control ZW gonads and AI-treated ZW gonads at stage 27. In AI-treated ZW embryonic gonads, Dmrt1 protein expression was strikingly up-regulated in medulla, as well as γH2AX protein expression almost disappeared. pre-sc, precursor sertoli cell; pgc, primordial germ cells. Scale bars are 50 μm.

Here, we analyzed the expression level of *Dmrt1* in gonads with sex reversal to further determine whether *Dmrt1* expression is associated with testis differentiation. qRT-PCR analysis showed that the mRNA expression of *Dmrt1* decreased dramatically in ZZ embryonic gonads during male to female sex reversal induced by E_2_. *Dmrt1* expression in feminized genetic males was down-regulated from stage 17, and reached its lowest expression level by stage 25 (Fig. 3a). Whereas in female to male sex reversed embryos after AI treatment, *Dmrt1* expression was induced from stage 18, peaked at stage 22 and was maintained at that high level (Fig. 3b). The higher level of *Dmrt1* expression in masculinized ZW gonads represented a >20-fold difference (versus that in control ZW gonads), implying active up-regulation of *Dmrt1* in males.

Stimulatory effects of AI or E_2_ treatment on *Dmrt1* expression were studied in more detail at the protein level. In mammals, female germ cells enter meiotic prophase where they remain until adulthood, whereas male germ cells arrest in G0/G1 phase of mitosis and do not enter meiosis until sexual maturity^29^. Here, no Foxl2 antibody is available to be successfully used in turtles and that, alternatively, we used meiosis-specific protein γH2AX as the embryonic ovarian marker. The dual immunofluorescence of Dmrt1 and γH2AX showed that in the control ZZ embryonic gonad at stage 27, Dmrt1 protein was abundantly expressed in the nuclei of precursor Sertoli cells, while no γH2AX protein signal was detectable in germ cells. Whereas, γH2AX protein was highly expressed in germ cells, and no Dmrt1 protein fluorescence signal was detected in control ZW embryonic gonads. In E_2_-treated ZZ embryonic gonads, the expression of Dmrt1 protein was almost completely lost, while γH2AX induction was localized in the gonadal cortex, exhibiting a female-like distribution pattern. In ZW embryonic gonads during male to female sex reversal induced by AI, Dmrt1 protein expression was strikingly up-regulated in the medulla, with γH2AX protein expression almost disappearing (Fig. 3c).

### Establishment of a lentivrius-mediated gene-modulating method in turtle embryos

**Figure 4:**
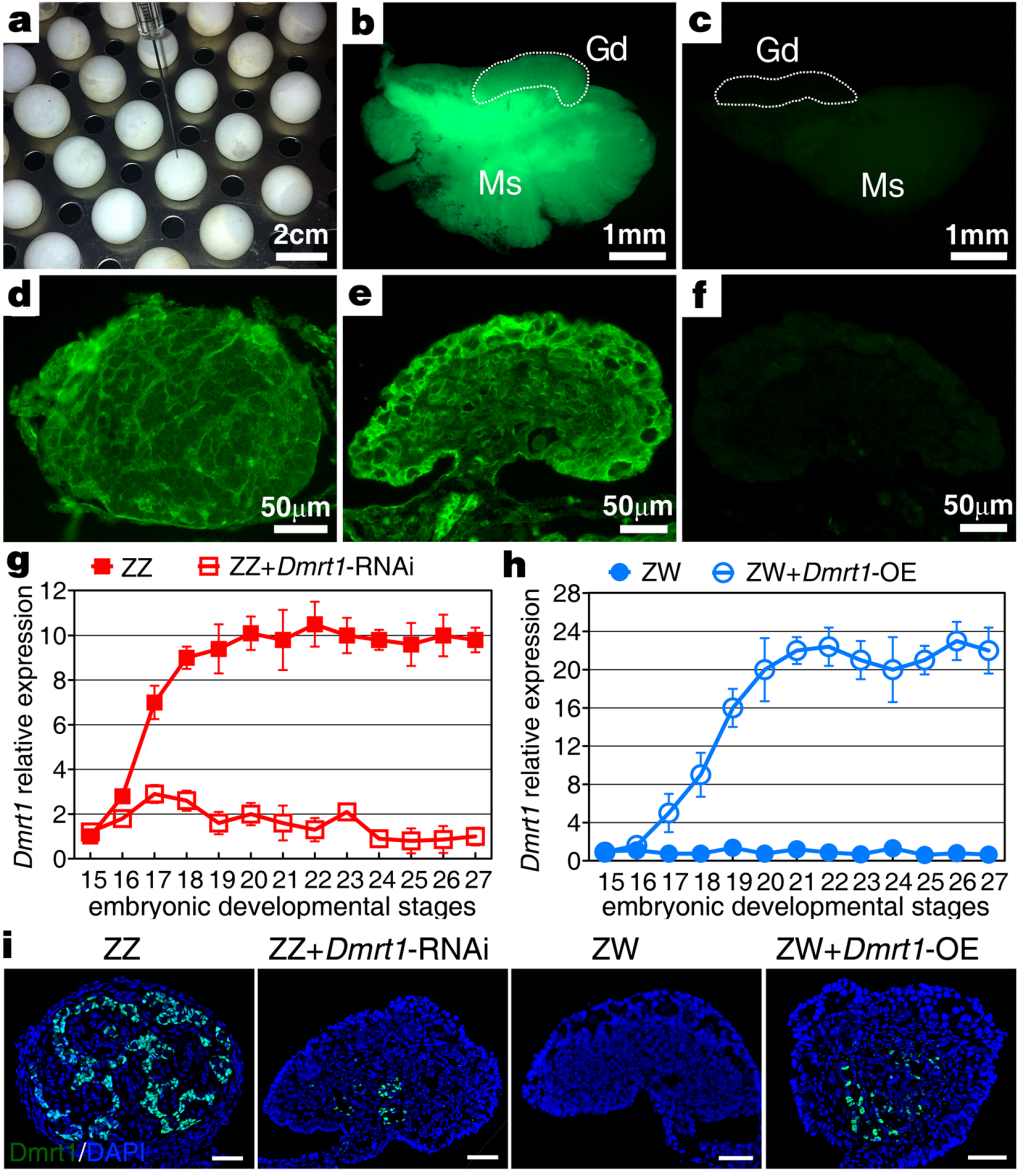
Establishment of a lentivrius mediated gene-modulating method in turtle embryos. (**a**) Chinese soft-shelled turtle embryos were injected with lentiviral vector at stage 14 before sexual differentiation, using a metal Hamilton needle. (**b**) Approximately 55% of embryos of stage 25 infected with the lentiviral vector showed robust and widespread GFP expression in gonad-mesonephros complexes (GMCs). (**c**) No GFP signals was seen in the negative control GMCs. Gd, gonad; Ms, mesonephros. (**d**,**e**) Robust GFP expression in sections of gonads treated with LV-*Dmrt1* or LV-*Dmrt1*-shRNA, determined by immunofluorescence. (**f**) No GFP expression were seen in the negative control gonadal sections. (**g**,**h**) The mRNA expression change of *Dmrt1* in gonads treated with LV-*Dmrt1*- shRNA or LV-*Dmrt1* was examined by qRT-PCR. Data are shown as means ± S.D. N ≥ 3. (**i**) The Dmrt1 immunofluorescence showed significantly down-regulation in ZZ gonad with LV-*Dmrt1*- shRNA treatment and ectopic expression in ZW gonad with LV-*Dmrt1*-OE treatment at stage 25, respectively. Scale bars are 50 μm.

In this study, lentiviral vectors carrying *Dmrt1* specific shRNAs or the ORF of *Dmrt1* were utilized to knockdown endogenous *Dmrt1* transcripts in ZZ gonads or induce ectopic expression of *Dmrt1* in ZW gonads, respectively. To test the efficacy of lentivirus delivery into the turtle embryos, GFP expression in turtle embryos injected with virus at stage 14 (Fig. 4a) were examined at stage 25. Approximately 55% of treated embryos showed global GFP reporter expression, including widespread expression in gonad tissues (Fig. 4b,c). GFP immunofluorescence of gonadal sections further confirmed that embryonic gonads with virus treatment exhibited robust GFP expression, indicating the effective infection of lentivirus in turtle gonadal tissues (Fig. 4d,e,f).

We then examined *Dmrt1* expression in embryonic gonads carrying LV-*Dmrt1*- shRNA (#1, Supplementary Fig. 4) or LV-*Dmrt1*-OE to figure out whether the lentivirus-mediated RNA interference or over-expression systems are able to efficiently modulate candidate genes expression. Here, genotype of all lentivirus-treated embryos with high GFP expression was determined by karyotype analysis and qPCR of 18S rRNA gene. qRT-PCR analysis showed that *Dmrt1* mRNA in genetic male gonads with LV-*Dmrt1*-shRNA was significantly reduced from stage 17, compared with that in control ZZ gonads (LV-NC-shRNA). The average inhibiting rate of *Dmrt1* mRNA expression by lentivirus-mediated RNA interference was 83.26% (Fig. 4g). In contrast, ectopic expression of *Dmrt1* mRNA in ZW gonads carrying LV-*Dmrt1*-OE was activated at stage 17 and sharply increased from stage 18, with the highest expression level exceeding 20-fold than that in the negative control ZW gonads (LV-empty) (Fig. 4h). Immunofluorescence analysis further revealed that in ZZ gonads with LV-*Dmrt1*-shRNA, no Dmrt1 protein expression was detected in the feminized cortical region, while the medullar region showed relatively weak Dmrt1 protein signals. In contrast, localized Dmrt1 protein expression was present in the medulla of ZW gonads with LV-*Dmrt1*-OE (Fig. 4i).

### Feminization of the ZZ gonads with *Dmrt1* knockdown

**Figure 5:**
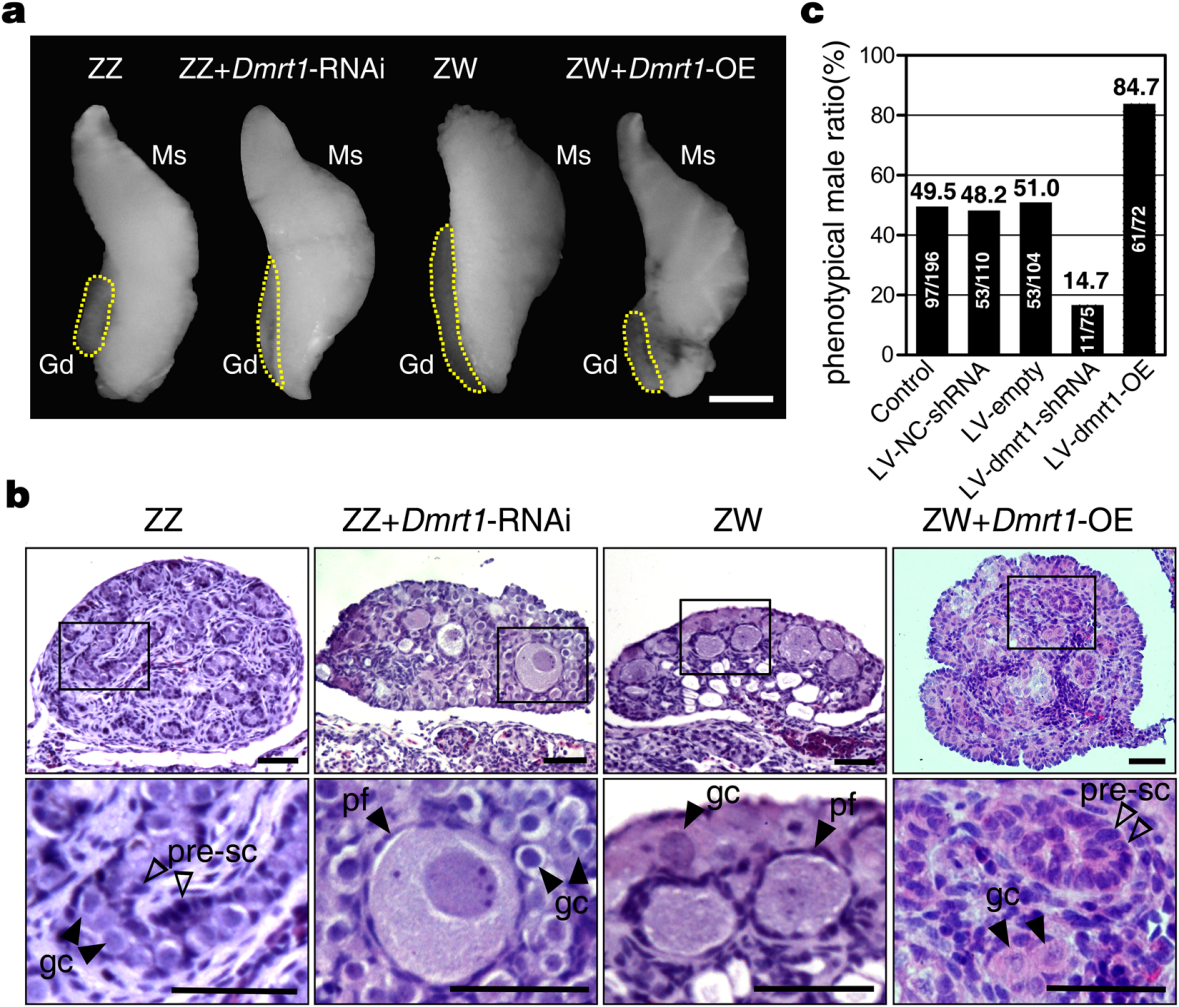
Effects of *Dmrt1* knockdown or over-expression on gonadal differentiation. (**a**) At stage 27, the ZZ gonads with *Dmrt1* knockdown became elongated and flat, compared to the control ZZ gonads. The ZW gonads overexpressing *Dmrt1* appeared distinctly short, similar to control ZZ gonads. Gd, gonad; Ms, mesonephros. Scale bar is 1 mm. (**b**) The H&E stain showed that thickened outer cortex with primordial follicles and degenerated testis cord were observed in the ZZ gonads with *Dmrt1* knockdown, however, the ZW gonads overexpressing *Dmrt1* exhibited a well developed medullary region with seminiferous cord-like structure. pre-sc, precursor sertoli cell; gc, germ cells; pf, primordial follicle. Scale bars are 50 μm. (**c**) The phenotypical male ratio of control embryos, scrambled control embryos with LV-NC-shRNA or LV-empty and embryos treated with LV-*Dmrt1*-shRNA or LV-*Dmrt1*-OE.

To investigate the function of *Dmrt1* on male development of *P*. *sinensis*, phenotype and marker genes expression of embryonic gonads with *Dmrt1* knockdown were assessed through gonadal histology, immunofluorescence and qRT-PCR. Gonadal development in embryos treated with non-silencing scramble shRNA or empty vector was normal. Control ZZ gonads were short and cylindrical, while ZW gonads were relatively long and flat. In ZZ embryos infected with LV-*Dmrt1*-shRNA and showing high GFP expression, gonads became elongated and flat, exhibiting varying degrees of female-like morphology (Fig. 5a). Histological analysis of gonadal sections by H&E staining showed that the control ZZ gonads of stage 27 had a dense medulla with seminiferous cords and a reduced cortex. Whereas the gonads of control ZW embryos possessed a well-developed outer cortex, populated with primordial follicles and a vacuolated medulla. In contrast, the *Dmrt1* knockdown ZZ gonads were strongly feminized, characterized by a thickened outer cortex with a large number of germ cells and a highly degenerated medulla (Fig. 5b). The statistic data showed that male proportion in the LV-*Dmrt1*-shRNA treated group was 14.7% (*vs*. 48.2% in controls) (Fig. 5c), and 71.8% (28 of 39) of ZZ gonads showed male to female sex reversal (Table 1).

**Table 1:** Phenotypes of embryos with knockdown or over-expression of *Dmrt1*.

Viral treatment	No. of injected embryos	Embryos surviving until stage 25	Embryos with high GFP expression	Genotype of embryos with high GFP	Phenotype of embryos with high GFP	sex reversal rate*
LV-NC-shRNA	250	197	110	ZZ:53; ZW:57	M:53; F:57	0/53
LV-*Dmrt1*-shRNA	300	141	75	ZZ:39; ZW:36	M:11; F:64	28/39
LV-empty	250	186	104	ZZ:53; ZW:51	M:53; F:51	0/51
LV-*Dmrt1*-OE	300	130	72	ZZ:38; ZW:34	M:61; F:11	23/34

Genotype of embryos with high GFP was determined by karyotype analysis and qPCR of 18S rRNA gene.

Phenotype of embryos was assessed by gonadal histology, immunofluorescent marker expression or qRT-PCR.

^*^Male to female sex reversal rate = No. of feminized genetic male embryos/total No. of ZZ embryos; female to male sex reversal rate = No. of masculinized genetic female embryos/total No. of ZW embryos.

**Figure 6:**
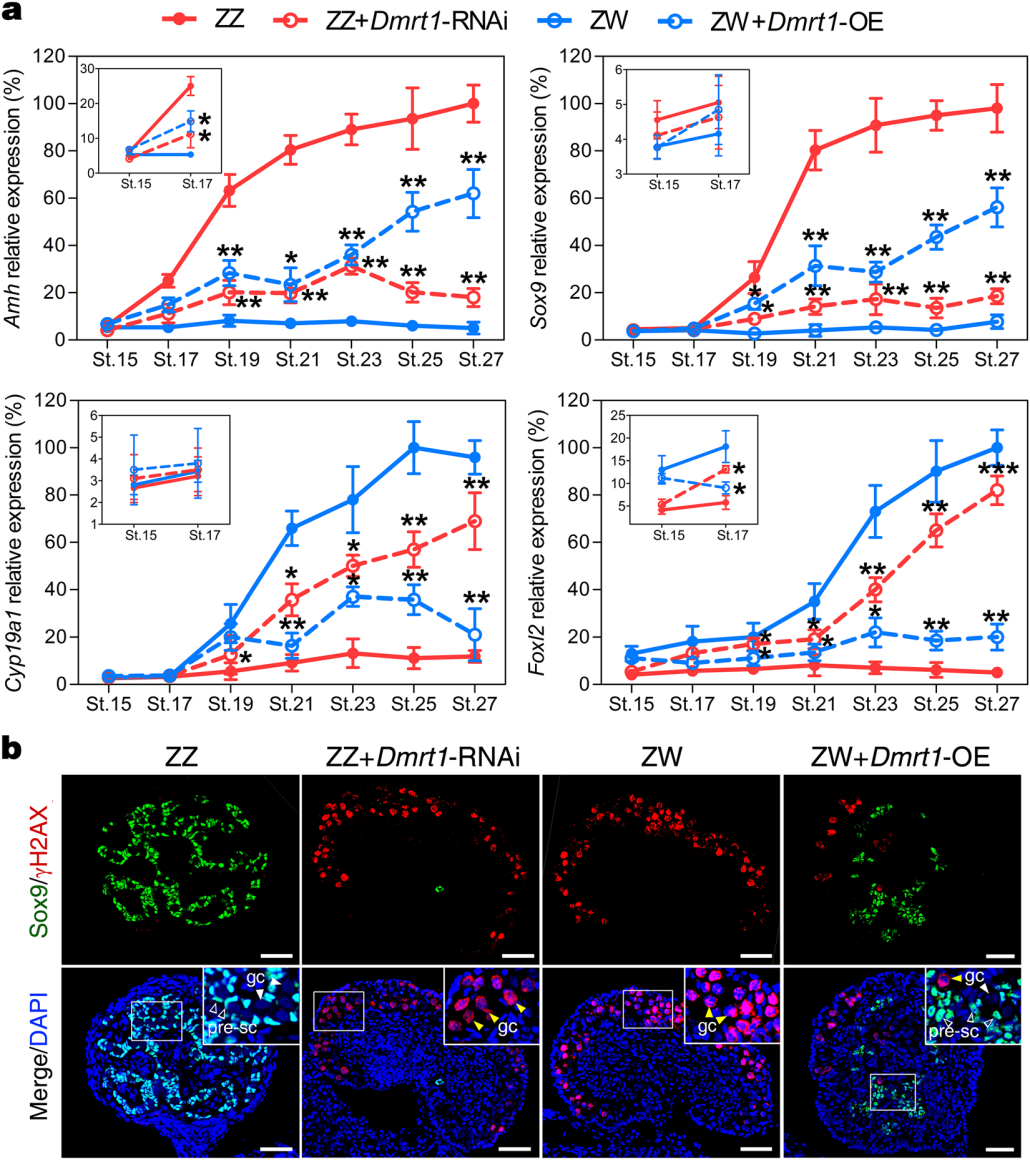
Responses of sex-specific genes to *Dmrt1* knockdown or over-expression. (**a**) qRT-PCR analysis showing the effects of *Dmrt1* knockdown or over-expression on the mRNA expression of *Amh*, *Sox9*, *Cyp19a1* and *Foxl2* in embryonic gonads at stage 15, 17, 19, 21, 23, 25 and 27. Data are shown as means ± S.D., N ≥ 3. *P < 0.05; **P < 0.01; ***P < 0.001. (**b**) Double immunofluorescence of Sox9 and γH2AX was performed in sections of control ZZ gonads, ZZ gonads with *Dmrt1* knockdown, the control ZW gonads and ZW gonads with *Dmrt1* over-expression at stage 27. pre-sc, precursor sertoli cell; gc, germ cells. Scale bars are 50 μm.

Male and female marker genes were examined at different embryonic stages to confirm the activation of the female developmental pathway in ZZ gonads with *Dmrt1* knockdown. At the mRNA level, remarkable down-regulation of testicular differentiation markers *Amh* and *Sox9*, and significant up-regulation of ovarian development regulators *Cyp19a1* and *Foxl2* were observed in ZZ gonads of different stages with *Dmrt1* knockdown relative to controls (Fig. 6a). *Amh* and *Foxl2* responded as early as stage 17 to *Dmrt1* knockdown, while *Sox9* and *Cyp19a1* showed a relative delayed response to *Dmrt1* knockdown, which occurred from stage 19 onward (Fig. 6a). At the protein level, Sox9 was expressed specifically in the nuclei of precursor Sertoli cells in control male gonads, while control female gonads lacked Sox9 expression. In ZZ gonads with *Dmrt1* knockdown, Sox9 expression almost disappeared; simultaneously germ cells in the feminized cortex were γH2AX positive, exhibiting a female-like distribution pattern (Fig. 6b).

**Figure 7:**
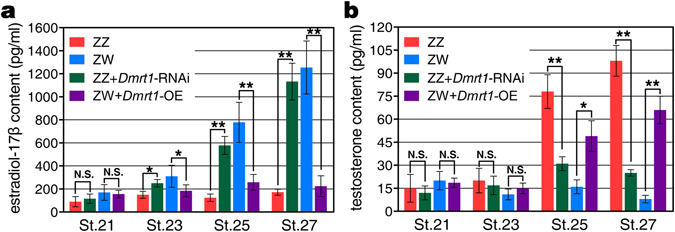
Effects of *Dmrt1* knockdown or over-expression on sex steroids production. Estradiol-17*β* (**a**) and testosterone (**b**) levels in control ZZ embryos, control ZW embryos, ZZ embryos injected with LV-*Dmrt1*-shRNA and ZZ embryos injected with LV-*Dmrt1* at different stages. Markedly increased estradiol-17*β* and significantly reduced testosterone levels in ZZ embryos with *Dmrt1* knockdown, demonstrated the female-biased steroid production in male to female sex reversal. In ZW embryos overexpressing *Dmrt1*, the opposite phenomenon were observed, showing the male-biased steroid production in female to male sex reversal. Data are shown as means ± S.D. N ≥ 3. *P < 0.05; **P < 0.01; N.S., no significance.

In vertebrates, sex-steroids play an important role in gonadal differentiation. Our above data showed that *Dmrt1* expression could be affected by exogenous estradiol-17β (E_2_) treatment (Fig. 3) and *Dmrt1* knockdown was able to regulate the expression of *Cyp19a1*, which encodes the key enzyme for estrogen synthesis (Fig. 6a). Therefore, it is necessary to analyze whether *Dmrt1* knockdown can alter steroid hormone production in turtle embryos. We measured the E_2_ and testosterone content in control and treated ZZ embryos at different stages (21, 23, 25 and 27). No significant difference was observed in E_2_ and testosterone production between control and *Dmrt1* knockdown ZZ embryos at stage 21. Up-regulation of E_2_ level in ZZ embryos with *Dmrt1* knockdown was observed from stage 23. This is consistent with *Cyp19a1* expression that had been remarkably increased in ZZ embryos with *Dmrt1* knockdown at this stage (Fig. 6a). At stages 25 and 27, significant differences in both E_2_ and testosterone levels were detected in control male and female embryos. *Dmrt1* knockdown in ZZ embryos resulted in up-regulation of E_2_ level and down-regulation of testosterone level (Fig. 7a,b).

### Masculinization of the ZW embryos over-expressing *Dmrt1*

To determine if *Dmrt1* was sufficient to initiate male development, we investigated the effect of ectopic expression of *Dmrt1* in genetic females. In ZW embryos over-expressing *Dmrt1*, gonads exhibited a short cylindrical structure, a feature specific to control ZZ gonads (Fig. 5a). Histological analysis for gonadal sections showed varying degrees of sex reversal phenotypes in ZW gonads over-expressing *Dmrt1*, including testes and ovotestes of variable proportions. The ovotestes exhibited a well-developed medulla with seminiferous cord-like structure and germ cells, as well as a partially degenerated cortex still containing germ cells (Fig. 5b). Testes and ovotestes were identified in 84.7% of virus treated embryos (Fig. 5c), and 67.6% (23 of 34) of genetic female embryos showed female to male sex reversal (Table 1).

Up-regulation of *Amh* and *Sox9* and down-regulation of *Cyp19a1* and *Foxl2* were observed in ZW gonads with *Dmrt1* over-expression, determined by qRT-PCR (Fig. 6a). However, these sex-specific genes differed in the earliest response time to *Dmrt1* over-expression, with the expression change of *Amh*, *Foxl2*, *Sox9* and *Cyp19a1* occurring from stages 17, 17, 19 and 21, respectively (Fig. 6a). Ectopic activation of Sox9 protein in treated ZW gonads was confirmed by immunofluorescence. Sox9 protein expression was induced and concentrated in the nuclei of Sertoli cells within the masculinized regions (testis cords) of ZW gonads over-expressing *Dmrt1*, but generally was lower and less widespread compared to control males. γH2AX protein expression was significantly decreased in some partially sex reversed ZW gonads, compared to that in female controls. However, a small number of γH2AX-positive meiotic germ cells still occurred in the cortial region (Fig. 6b). Co-existence of Sox9 and γH2AX further confirmed the partial masculinization of ZW gonads, consistent with the histological analysis results. For sex steroids, over-expression of *Dmrt1* in ZW embryos resulted in significant down-regulation of E_2_ production and markedly increased testosterone levels at stage 25 and 27 (Fig. 7a,b).

## Discussion

In the present study, we provide solid evidence that *Dmrt1* gene is both necessary and sufficient for primary testicular differentiation in Chinese soft-shelled turtle, through a newly developed lentivrius-mediated gene-modulating approach.

In *P*. *sinensis*, specific expression of *Dmrt1* has already appeared in male gonads as early as stage 16, prior to the onset of gonadal sex differentiation, which occurs at stage 17. Subsequently, robust and consecutive expressions of *Dmrt1* followed by *Amh* and *Sox9*, were detected only in the male gonads throughout embryogenesis. This dimorphic expression pattern indicates the possible involvement of *Dmrt1* in primary male sex determination. These results are consistent with previous studies in red-eared slider turtle, *Trachemys scripta*, that reported *Dmrt1* mRNA expression in early male embryos was higher than that in females, preceding *Sox9* expression^30,31^. In non-mammalian vertebrates, estrogen plays an important regulatory role in early gonadal sex differentiation, while exogenous estrogen can significantly suppress *Dmrt1* expression in male embryos^23,32,33^. In *T*. *scripta*, *Dmrt1* mRNA expression was significantly inhibited in estrogen-treated male embryos, while in female embryos with AI treatment, *Dmrt1* expression was dramatically up-regulated^34,35^. In this study, we found that *Dmrt1* expression was significantly reduced in feminized ZZ embryos of *P*. *sinensis* treated with E_2_, whereas remarkable up-regulation of *Dmrt1* expression was observed in the distinctly masculinized ZW embryos of *P*. *sinensis* induced by AI, implying that *Dmrt1* associated with male gonadal differentiation of *P*. *sinensis*, and repression of *Dmrt1* by estrogen signaling is highly conserved in non-mammalian vertebrates, although this process may be indirect. These observations suggest that *Dmrt1* gene participates in male sexual development of *P*. *sinensis*, likely located upstream of the male pathway.

To date, the complete analysis of *Dmrt1* function in sexual differentiation and testicular development has never been performed in reptiles, including turtles, largely due to the unavailability of gene knockout and transgenic techniques. Recently, a number of studies have been focusing on the novel establishment of effective gene-modulating methods in turtle species. Itzel *et al*.^36^ reported a 70–80% reduction of *Sox9* mRNA and protein expression in *in vitro* cultured male gonads of Olive ridley sea turtles, *Lepidochelys olivacea*, by using RNA interference. In contrast, GFP: Sox9 expression vector was introduced into *in vitro* female cultured gonads of red-eared slider turtles by electroporation, resulting in a certain amount of ectopic *Sox9* expression^37^. However, no *in vivo* genetic manipulation methods were achieved in turtle embryos. In this study, high-titre lentiviral vectors carrying *Dmrt1* specific shRNA or *Dmrt1* ORF were injected into *P*. *sinensis* embryos at stage 14, leading to the global GFP reporter expression in 55% of treated embryos at stage 25, as well as efficient knockdown of *Dmrt1* in ZZ gonads (inhibiting rate >80%) and robust over-expression of *Dmrt1* in ZW gonads (over 20 times higher) throughout the embryogenesis of *P*. *sinensis*. This lentiviral vector expression system showed obvious infection of turtle embryos and *in vivo* efficient gene-modulating ability, which provides a powerful tool for gene functional analysis in turtle species.

Using a novel method of genetic manipulation in *P*. *sinensis* embryos, we revealed that *Dmrt1* knockdown in ZZ embryos caused complete feminization of male gonads, with down-regulation of testicular markers *Amh* and *Sox9* and up-regulation of ovarian regulators *Cyp19a1* and *Foxl2*. Moreover, ZZ gonads with *Dmrt1* knockdown exhibited γH2AX positive meiotic germ cells in the thickened outer cortex, which only occurred in female gonads. These observations are quite similar with that in other vertebrates. In ZZ chick embryos, downregulation of *Dmrt1* by RNA interference led to the feminization of embryonic gonads, a decline in *Sox9* expression and an increase in *Cyp19a1* and *Foxl2* expression^9^. *Dmy* knockdown in XY medaka larvae caused increased number of meiotic germ cells, suppressed the male pathway (*Gsdf*, *Sox9a2*, etc.) and favored the female cascade (*Rspo1*, *Foxl2*, etc.), resulting in a fertile male to female sex reversal^38,39^. Most importantly, in the converse experiment, *Dmrt1* over-expression in ZW gonads of *P*. *sinensis* triggered the formation of sex cord-like structures, activated the ectopic expression of *Amh* and *Sox9*, and antagonized *Cyp19a1* and *Foxl2* expression, suggesting that *Dmrt1* is capable of initiating the male developmental pathway. Likewise, over-expression of *Dmy* in XX medaka resulted in testicular differentiation^40^. It is known that *Dmy* can activate expression of autosomal *Dmrt1*, which is involved in testicular differentiation as a male sex maintainer. Furthermore, evidence has shown that *Dmrt1* mutations cause male to female sex reversal after the sex is determined by *Dmy* in the medaka^41–43^. In chick embryos, ZW gonads over-expressing *Dmrt1* exhibited a masculinized morphology, as well as activation of *Amh* and *Sox9*^10^. These findings demonstrated that *Dmrt1* is both necessary and sufficient to initiate male development in *P*. *sinensis*. In addition, *Amh* and *Sox9* were upregulated following over-expression of *Dmrt1* in ZW gonads, suggesting that ectopic expression of these two genes may be a response to elevated *Dmrt1*. Based on this expression pattern, implication arises that *Dmrt1* lies upstream of *Amh* and *Sox9* in *P*. *sinensis*. Taken together, all these observations suggest that *Dmrt1* exerts a key upstream regulator role in primary male sexual differentiation of *P*. *sinensis*, although it is uncertain whether a Z-linked gene exists upstream of *Dmrt1*, acting as the male sex-determining gene. Of course, it cannot be excluded that a W-linked gene functions as the female sex-determining gene in *P*. *sinensis*, similar to *Dmw* in *Xenopus laevis*^11,12^. Thus further research is needed to elucidate the full sex-determining mechanism of *P*. *sinensis*.

In summary, we demonstrate here for the first time that *Dmrt1* is not only necessary for primary male sexual differentiation, but also sufficient to trigger testicular development in *P*. *sinensis*, thereby acting as an upstream regulator of the male pathway. In addition, the lentivirus-mediated genetic manipulation approach worked well in turtle embryos, which provides a powerful foundation for the functional studies of interesting genes in turtle species.

## Methods

### Eggs Incubation and Tissue Collection

Freshly laid Chinese soft-shelled turtle eggs were obtained from the Dafan turtle farm (Zhejiang, China). Fertilized eggs were incubated in an egg incubator at 31 °C, with humidity maintained at 75–85%. During the process of egg incubation, embryos of different developmental stages: stage 14 (14 days), stage 15 (16 days), stage 16 (18 days), stage 17 (20 days), stage 18 (22 days), stage 19 (24 days), stage 20 (26 days), stage 21 (28 days), stage 22 (31 days), stage 23 (34 days), stage 24 (37 days), stage 25 (40 days), stage 26 (43 days), stage 27 (48 days; hatching), which were distinguished according to previous reports in turtles^44–46^, were removed from eggshells, decapitated, and placed in PBS for dissection. Gonad-mesonephros complexes (GMCs) and whole-gonads adjacent to mesonephros were dissected from treated and control embryos, respectively. GMCs were fixed in 4% paraformaldehyde (PFA) overnight at 4 °C, dehydrated through a methanol gradient, and stored in 70% ethanol at 4 °C until paraffin embedding and sectioning was performed. Other gonads were broken up thoroughly and stored in TRIzol reagent (Invitrogen, USA) for total RNA isolation. Meanwhile, these embryos were treated by liquid nitrogen grinding and then stored at −80 °C for genomic DNA extraction. Additionally, the tissue samples of heart, liver, spleen, lung, kidney, muscle, brain, testis and ovary from adult turtles were prepared and stored at −80 °C for cDNA cloning and expression analysis. Metaphase chromosome spreads of *P*. *sinensis* were prepared from fibroblast cells for karyotype analysis according to previous studies in our laboratory^47^ (Supplementary Fig. 5). All animal experiments were carried out following the guidelines approved by the Institutional Animal Care and Use committee at Zhejiang Wanli University, China.

### Cloning of *P*. *sinensis Dmrt1* cDNA

The total RNA from testis of adult turtle *P*. *sinensis* was extracted using TRIzol reagent (Invitrogen, USA), and then treated with DNase I (Takara, Japan). The first complementary DNA (cDNA) was then synthesized from 2 μg of RNA by using the RevertAid^™^ First Strand cDNA Synthesis Kit (Fermentas, USA) according to the manufacturer’s instructions. Gene-specific primers were designed based upon the published partial sequence of *P*. *sinensis Dmrt1* (accession No. XP_006137928.1). 5′ RACE and 3′ RACE were carried out according to the manufacturer’s protocol of SMART RACE cDNA Amplification kit (Clontech, Takara, Japan). The gene-specific primers (*Dmrt1*-F1: 5′-GTCAAGCCAGTCAGGAAACCAG-3′; *Dmrt1*-F2: 5′-GGACGGATGCTCATTCAGGACA-3′; *Dmrt1*-F3: 5′-TAGGGACCATAGC TTCAC-3′; *Dmrt1*-R1: 5′-GGCTGCTGCTTTCCAACAATAA-3′; *Dmrt1*-R2: 5′-GCTGCC TTCTCAATGCAACCTG-3′; *Dmrt1*-R3: 5′-TCGGCTGGTTCGCCTCTACAAT-3′; *Dmrt1*- R4: 5′-TTGCTCCGATGAGACCCAAGTAA A-3′) were used for RACE. The PCR products were extracted from agarose gel using MiniBEST Agarose Gel DNA Extraction Kit (Takara, Japan) based on manufacturer’s instructions, and cloned into pMD20-T (Takara) vector and then transformed into *E*.*coli* DH5α for sequencing.

Alignment of deducted amino acid sequences were carried out by Clustal X software and MegAlign program in DNAStar software, while the phylogenetic tree was constructed using the Neighbour-Joining(N-J) method in Mega 6.0 software. The values on the tree represent bootstrap scores of 1,000 trials, indicating the credibility of each branch. The sequences of nucleotide and amino acid used in the phylogenetic analysis were obtained from GenBank (NCBI).

### Estradiol-17β and Aromatase Inhibitor Treatments

Estradiol-17β (E_2_, Sigma, USA) or a non-steroidal aromatase inhibitor (AI) letrozole (Sigma) were administered to eggs before sexual differentiation. The E_2_/AI was dissolved in 95% ethanol at a concentration of 20 μg/μl, and 5 μl of drug was topically applied to the eggshell in the region adjacent to the embryo at developmental stages 15 and 16 (gonadal differentiation normally begins at stage 17). Controls were treated with 5 μl of 95% ethanol. Egg incubations for both treated and control groups were performed under the same condition as previously described. Gonad-mesonephros complexes were dissected from treated and control embryos at stage 25 for histology and immunohistochemistry use. Gonads were separated from adjacent mesonephros at stage 16, 17, 18, 19, 20, 21, 22, 23, 24, 25, 26 and hatching time, and preserved for qRT-PCR analysis.

### Construction of LV-*Dmrt1*-shRNA Vector System

Three shRNAs targeting turtle *Dmrt1* mRNA were designed to give rise to siRNA, using the shRNA designer website (http://rnaidesigner.thermofisher.com/rnaiexpress/design.do). The lentivirus vector was used to deliver shRNAs directed specifically against turtle *Dmrt1* mRNA into living embryos of Chinese soft-shell turtle before sexual differentiation, to knockdown endogenous *Dmrt1* transcripts. The designed shRNA construct contained a unique 21 nt double-stranded *Dmrt1* sequence that presented as an inverted complementary repeat, a loop sequence (5′-CTCGAG-3′) and the RNA PloIII terminator (5′-TTTTTT-3′). Annealed oligonucleotides were ligated into pGP-U6 (GenePharma, Shanghai, China) between the *Bbs* and *Xho* sites by T4 DNA ligase (TaKaRa) to produce pGP-U6-*Dmrt1*-shRNA. The pGP-U6-*Dmrt1*-shRNA construct was digested with *Age*I-*EcoR*I and inserted into the *EcoR*I site of pGLV-U6-GFP (GenePharma). The lentivirus vector can also express green fluorescent protein (GFP), providing rapid visual assessment of the viral infection efficiency of embryos. The recombinant vector pGLV-GFP-*Dmrt1*-shRNA was termed as LV-*Dmrt1*- shRNA. The negative control vector (pGLV-GFP-NC-shRNA, termed as LV-NC-shRNA) contained a nonsense shRNA insert in order to control any effects caused by non-RNAi mechanisms. The sequences of the shRNAs are as follows: *Dmrt1*-shRNA#1(5′-GGTGGCAGCTCCTGTTTATTG-3′); *Dmrt1*-shRNA#2(5′-GGATGCTCATTCAGG ACATTC-3′); *Dmrt1*-shRNA#3(5′-GCAGTCAAGACTCTGGCTTAA-3′); negative control (5′-TTCTCCGAACGTGTCACGTAT-3′).

For the generation of lentivirus, 293 T producer cells were transfected with optimized packaging plasmids (pGag/Pol, pRev and pVSV-G) along with pGLV-*Dmrt1*-shRNA or pGLV-NC-shRNA expression clone constructs by lipofectamine. 24 h post transfection, the transfection mix was replaced by a fresh culture medium (without antibiotics). The virus-containing supernatant was harvested 72 h post transfection, cleared by centrifugation (3000 rpm/min, 15 min, and 4 °C), and then filtered through a 0.45 μm filter (Millipore, USA). Viruses were titrated by adding serial dilutions to fresh 293 T, and assessed using GFP expression after 48 h. Viral titres of approximately 1 × 10^9^ infectious units/ml were obtained. Lentivirus aliquots were stored at −80 °C before infection of turtle embryos.

### Construction of LV-*Dmrt1*-OE Vector System

Total RNA isolation was performed on the testis of adult Chinese soft-shelled turtle and then followed by reverse transcription to prepare the cDNA (methodology found above). Based on the complete cDNA sequence of *P*. *sinensis Dmrt1* gene, the open reading frame (1107 bp) was PCR amplified from cDNA using forward primer 5′-CCCCAAATTGTAGAGGCGAACC-3′ and reverse primer 5′-TGAGGGCAGGGCAGAGGAGG-3′. The PCR product was digested with *EcoR*I and cloned to pGLV-EF1a-GFP (LV-4, GenePharma). The recombinant vector pGLV-GFP-*Dmrt1* was named LV-*Dmrt1-OE*. The empty vector pGLV-GFP-empty was considered as a negative control (LV-empty). High quality proviral DNA was then used to transfect 293 T cells. Virus propagation was carried out as described above.

### Infection of Turtle Embryos

High titre virus of LV-*Dmrt1*-shRNA or LV-*Dmrt1* (at least 1 × 10^9^ infectious units/ml) was injected into turtle embryos at stage 14, using a metal Hamilton needle(the diameter of the needle: 0.5 mm). Approximately 5 μl was injected per embryo, and a total of 500 eggs were injected in every treated group. Every 300 control embryos were injected with scrambled control virus of LV-NC-shRNA or LV-empty. Eggs were sealed with parafilm and incubated for the indicated time points (stage 15 to 27). Embryos showing GFP fluorescence in the urogenital system were chosen for further analysis.

### Quantification of 18S rRNA Repeat Copy Number

Copy number of the 18S rRNA repeats was quantified in each embryo by qPCR to identify the individual sex except using karyotype analysis in this study. This examination was performed as previously described^48^. The genomic DNA was first extracted from all tested embryos, and subsequently copy number of the 18S gene was normalized against GAPDH using the comparative Ct method of normalization (Ratio (18 S/GAPDH) = 2^Ct GAPDH − Ct 18S^). A higher value of ΔCt (GAPDH - 18S) indicates a larger number of copies of 18S rRNA repeats. The sequences of primers are as follows: 18S rRNA (F: 5′-GAGTATGGTTGCAAAGCTGAAA-3′, R: 5′-CGAGAAAGAGCTATCAATCTGT-3′); GAPDH (F: 5′-GGCTTTCCGTGTTCCAACTC-3′, R: 5′-GACAACCTGGTCCTCCGTGTATC-3′). The W chromosome in *P*. *sinensis* has a much larger number of copies of rRNA genes compared to the Z chromosome according to FISH mapping results^49^, thus female individuals have more copies of 18S rRNA genes than males (Supplementary Fig. 6).

### Quantitative RT-PCR

Total RNA extraction was performed on various adult tissues and embryonic gonads at different developmental stages in all treated and control groups, and subsequently synthesized for cDNA. The mRNA expression levels for different adult turtle tissues were examined by semi-quantitative RT-PCR, and *Gapdh*(F: 5′-GGCTTTCCGTGTTCCAACTC-3′, R: 5′-GACAACCTGGTCCTCCGTGTATC-3′) was used as a reference gene. Quantification of transcript levels was measured by quantitative RT-PCR(qRT-RCR). The reaction was performed with SYBR^®^ PrimeScript^TM^ II (Takara) in a Bio-Rad iCycler system. After normalization with *β*-*actin*, relative RNA levels in samples were calculated using the comparative threshold cycle (Ct) method. Each RNA sample was analyzed in triplicate determinations. The sequences of primers for PCR are as follows: *β-actin* (F: 5′-AACTGGGATGACATGGAGAAGA-3′, R: 5′-AACATGATCTGGGTCATCTT-3′); *Dmrt1* (F: 5′-CCGCCTCGGGAAAGAAGTC-3′, R: 5′-TGCTGGATGCCGTAGTTGC-3′); *Amh* (F: 5′-CGGCTACTCCTCCCACACG-3′, R: 5′-CCTGGCTGGAGTATTTGACGG-3′); *Sox9* (F: 5′-AGCCTCTATTCCACCTTCAC-3′, R:5′-ATGTCTGTACCGAGTTTTGC-3′); *Cyp19a1* (F: 5′-TCGTGGCTGTACAAGAAATACGAA-3′, R: 5′-CCAGTCATATCTCCACGGCTCT-3′); *Foxl2* (F: 5′-GCGGACGTCCTTCTCTCC-3′, R: 5′-GACACACACAGACGGCTGG-3′).

### Western Blot Analysis

Total protein extraction from different tissues of adult Chinese soft-shelled turtle was extracted with RIPA lysis buffer (Santa Cruz, USA). Western blot was performed to examine the Dmrt1 protein expression in adult tissues. Equal amounts of denatured protein samples were separated on 10% SDS-PAGE (sodium dodecyl sulfonate-polyacrylamide gel electrophoresis) and transferred onto a PVDF (polyvinylidene difluoride) membrane. The membrane was incubated in 5% dry skim milk at room temperature for 1 h and subsequently with primary antibody (rabbit anti Dmrt1) at dilutions recommended by the supplier (dilution 1:1000, Millipore, USA) overnight at 4 °C. Antibody recognition was detected with the secondary antibody linked to horseradish peroxidase (goat anti-rabbit IgG-HRP, 1:5000, Santa Cruz, USA) at room temperature for 60 min. β-actin bands were used as an internal control. The immunoreactive bands were visualized with DAB (3,3N-Diaminobenzidine Tertrahydrochloride) Horseradish Peroxidase Color Development Kit (Beyotime, china).

### Immunofluorescence

GMCs and gonad tissues (testis or ovary) of adult Chinese soft-shelled turtle were fixed in 4% PFA at 4 °C, dehydrated, and then embedded in paraffin sections. Paraffin sections (5–6 μm) were deparaffinized and rehydrated in graded ethanol. Histological changes were observed on treated gonads by hematoxylin and eosin (H&E) staining. Additionally, immunofluorescence staining was carried out to analyze the expression changes of proteins. Antigen retrieval was performed in 10 mM sodium citrate buffer for 15 min at a sub-boiling temperature (99 °C). Sections were then blocked for 60 min in blocking solution (10% Normal Donkey Serum, 3% BSA(albumin from bovine serum), and 0.3% Triton X-100) at room temperature, followed by primary antibodies incubation (overnight, 4 °C), washing (three times), secondary antibodies incubation (60 min, room temperature, dark environment) and washing (three times). Washing was performed for 10 min each time in washing solution (1% Normal Donkey Serum, 3% BSA, 0.3% Triton X-100). The primary antibodies used in this analysis included rabbit anti Dmrt1(1:250, Millipore), rabbit anti-Sox9 (1:500, Millipore), rabbit anti-GFP (1:250, Abcam, Britain), mouse anti-CTNNB1 (1:250, Sigma) and mouse anti-γH2AX (1:500, Abcam). Primary antibodies were detected using secondary antibodies (AlexFluor 488 donkey anti-rabbit IgG, AlexFluor 594 donkey anti-mouse IgG, 1:250, Invitrogen). Nuclei were stained with DAPI (4′, 6-diamidino-2-phenylindole, 286 nmol/L, Sigma) and then washed with 0.01 mol/L PBS (phosphate buffered saline) three times (5 min each time). Gonad sections were imaged under a fluorescence microscope (Ti-E, nickon) or confocal microscope (A1 Plus, Nickon).

### Steroids Measurement

Steroid was extracted from the control ZZ/ZW embryos, ZZ embryos injected with LV-*Dmrt1*-shRNA, and ZW embryos injected with LV-*Dmrt1* at stage 21, 23, 25, and 27 respectively. Blood collection was first performed from one hundred de-yolked embryos in each group and then was quickly diluted in 5% sodium citrate solution. The blood samples were stored at −80 °C for steroid measurement. The estrogen and testosterone concentrations were measured in each sample, using the estradiol-17β (E_2_) and testosterone high sensitivity ELISA kit (Enzo, Japan) based on manufacturer’s instructions, respectively. Steroid content was calculated according to the standard curve drew using respective steroids, provided by the manufacturer (Enzo).

### Statistical Analysis

Each experiment was independently repeated at least 3 times. All data was expressed as the means ± S.D. and analyzed by ANOVA and Duncan’s multiple range tests using the SAS 6.12 software. For all analyses, a *P*-value < 0.05 was regarded as statistically significant.

## Electronic supplementary material

supplementary information
