# Author Correction: *Dmrt1* is required for primary male sexual differentiation in Chinese soft-shelled turtle *Pelodiscus sinensis*

**DOI:** 10.1038/s41598-018-23722-7

**Published:** 2018-04-17

**Authors:** Wei Sun, Han Cai, Gloria Zhang, Haiyan Zhang, Haisheng Bao, Li Wang, Jian Ye, Guoying Qian, Chutian Ge

**Affiliations:** 1 College of Biological and Environmental Sciences, Zhejiang Wanli University, Ningbo, 315100 China; 2 Trinity School of Arts and Sciences, Duke University, Durham, NC 27708 USA; 3 College of Fisheries and Life Sciences, Shanghai Ocean University, Shanghai, 201306 China; 4 HangZhou Aquacultural Technique Extending Centre, Hangzhou, 310001 China

Correction to: *Scientific Reports* 10.1038/s41598-017-04938-5, published online 30 June 2017

Some of the data presented in this Article was published by the Authors previously. The Authors neglected to cite this previous paper, which is included below as ref.^1^ and should be cited in legend of Figure 6 as below:

“Figure 6. Responses of sex-specific genes to *Dmrt1* knockdown or over-expression. (**a**) qRT-PCR analysis showing the effects of *Dmrt1* knockdown or over-expression on the mRNA expression of *Amh*, *Sox9*, *Cyp19a1* and *Foxl2* in embryonic gonads at stage 15, 17, 19, 21, 23, 25 and 27. Data are shown as means  ±  S.D., N  ≥  3. *P  <  0.05; **P  <  0.01; ***P  <  0.001. (**b**) Double immunofluorescence of Sox9 and γH2AX was performed in sections of control ZZ gonads, ZZ gonads with *Dmrt1* knockdown, the control ZW gonads and ZW gonads with *Dmrt1* over-expression at stage 27. pre-sc, precursor sertoli cell; gc, germ cells. Scale bars are 50 μm.”

should read:

“Figure 6. Responses of sex-specific genes to *Dmrt1* knockdown or over-expression. (**a**) qRT-PCR analysis showing the effects of *Dmrt1* knockdown or over-expression on the mRNA expression of *Amh*, *Sox9*, *Cyp19a1* and *Foxl2* in embryonic gonads at stage 15, 17, 19, 21, 23, 25 and 27. Data are shown as means  ±  S.D., N  ≥  3. *P  <  0.05; **P  <  0.01; ***P  <  0.001. Data for ZZ was published before in ref.^1^ as Figure 3C and is reprinted here with permission. (**b**) Double immunofluorescence of Sox9 and γH2AX was performed in sections of control ZZ gonads, ZZ gonads with *Dmrt1* knockdown, the control ZW gonads and ZW gonads with *Dmrt1* over-expression at stage 27. pre-sc, precursor sertoli cell; gc, germ cells. Scale bars are 50  μm.
