# Supplementary material for: Dmrt1 is required for primary male sexual differentiation in Chinese soft-shelled turtle Pelodiscus sinensis
Source: Sci Rep. 2017 Jun 30;7:4433. doi: 10.1038/s41598-017-04938-5 (PMC5493664; doi:10.1038/s41598-017-04938-5)
Supplement: Supplementary file 1 — supplementary information [file 41598_2017_4938_MOESM1_ESM.pdf]

SUPPLEMENTARY INFORMATION

Dmrt1* is required for primary male sexual differentiation in Chinese soft-shelled turtle, *Pelodiscus sinensis

Wei Sun[a,1], Han Cai[a,1], Gloria Zhang[b], Haiyan Zhang[a], Haisheng Bao[c], Li Wang[c], Jian Ye[d], Guoying Qian[a, 2], Chutian Ge[a, 2]

[a]College of Biological and Environmental Sciences, Zhejiang Wanli University, Ningbo, 315100, China

[b]Trinity School of Arts and Sciences, Duke University, Durham, NC 27708, USA

[c]College of Fisheries and Life Sciences, Shanghai Ocean University, Shanghai, 201306, China

[d]HangZhou Aquacultural Technique Extending Centre, Hangzhou, 310001, China

[1] These authors contributed equally to this work.

[2] Corresponding authors:

Guoying Qian, Ph.D., College of Biological and Environmental Sciences, Zhejiang Wanli University, Ningbo 315100, P. R. China. Tel: 86-574-88222298; e-mail: qiangy@zwu.edu.cn;

Chutian Ge, Ph.D., College of Biological and Environmental Sciences, Zhejiang Wanli University, Ningbo 315100, P. R. China. Tel: 86-574-88223277; e-mail: cge@zwu.edu.cn.

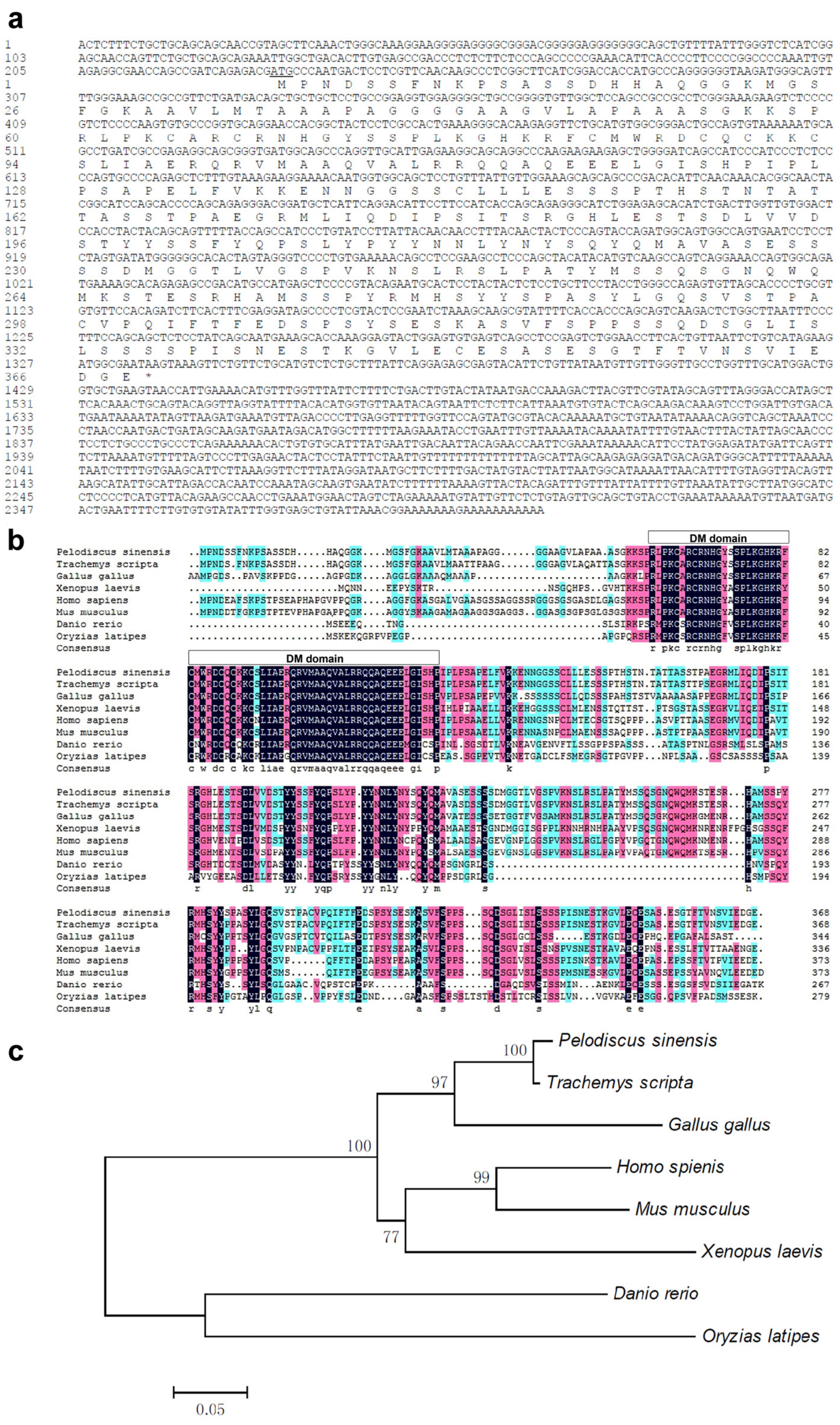

Figure S1. Sequence and phylogenetic analyses of ***P. sinensis*** ***Dmrt1*****.** (a) The complete cDNA sequence of *P. sinensis Dmrt1* and deduced amino acid sequence. The start codon ATG was underlined, and the stop codon was indicated by an asterisk. (b) Alignment of amino acid sequence of *P. sinensis* Dmrt1 with those from other typical species. The highly conserved DM domain were marked. (c) Dmrt1 phylogenetic tree from *P. sinensis* and other typical species based on Neighbor-Joining (N-J) method. Numbers at branches were confidence values based on 1000 bootstraps. Each branch length scale in terms of genetic distance was indicated above the tree.

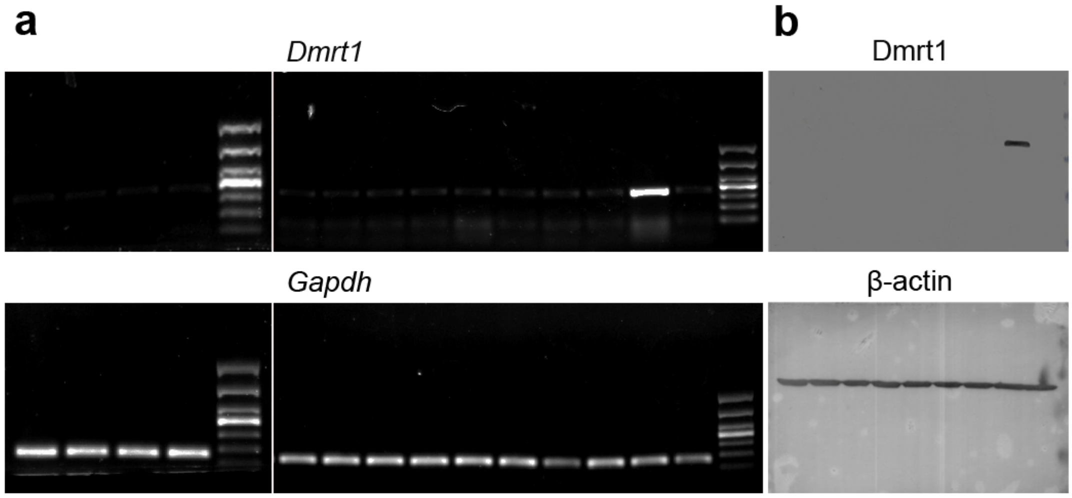

Figure S2. The testis-specific expression of *Dmrt1* in *Pelodiscus sinensis*. (a) The expression of *Dmrt1* mRNA in different tissues were analyzed by RT-PCR. (b) The expression of Dmrt1 protein in different tissues was examined by western blot.

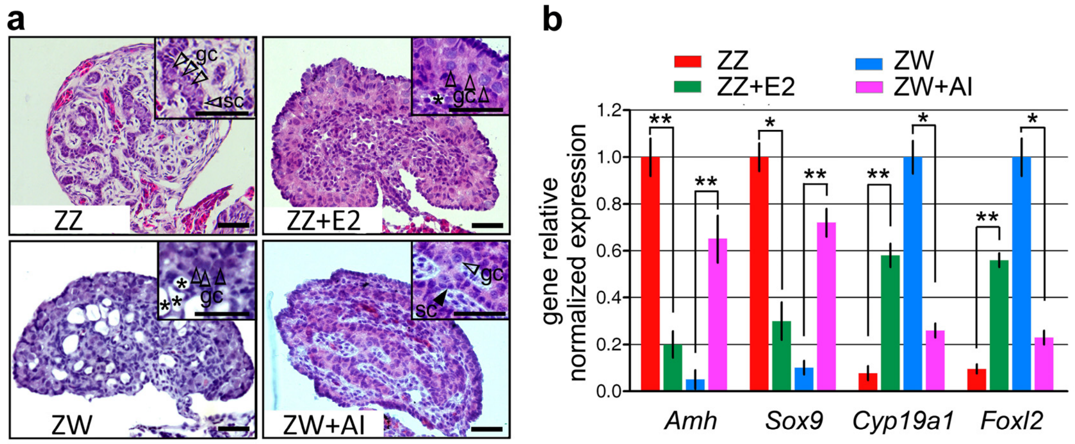

Figure S3. Effects of E_2 and AI on sexual differentiation. (a) H&E staining showed that obvious feminization of ZZ gonads and masculinization of ZW gonads at stage 27 were observed after E_2 and AI treatment, respectively. sc, sertoli cell; gc, germ cells. Scale bars are 50 μm. (b) qRT-PCR demonstrated that *Amh* and *Sox9* expression were significantly down-regulated, and *Cyp19a1* and *Foxl2* expression were dramatically up-regulated in ZZ gonads after E_2 treatment. AI exerted the opposite effect in ZW gonads. Data are shown as means ± S.D. *, $P < 0.05$; **, $P < 0.01$.

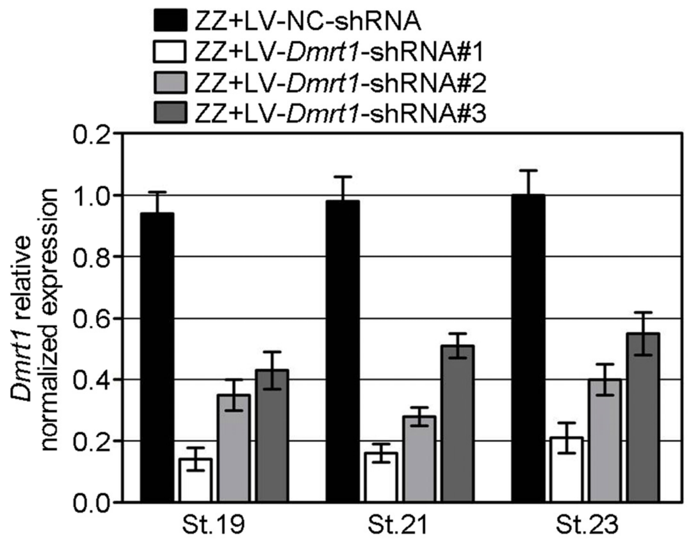

Figure S4. Interference efficiency of three pairs of shRNAs. qRT-PCR demonstrated that the interference efficiency of *Dmrt1*-shRNA#1 was highest between the three pairs of shRNAs (#1, 2, 3) at stage 19, 21 and 23.

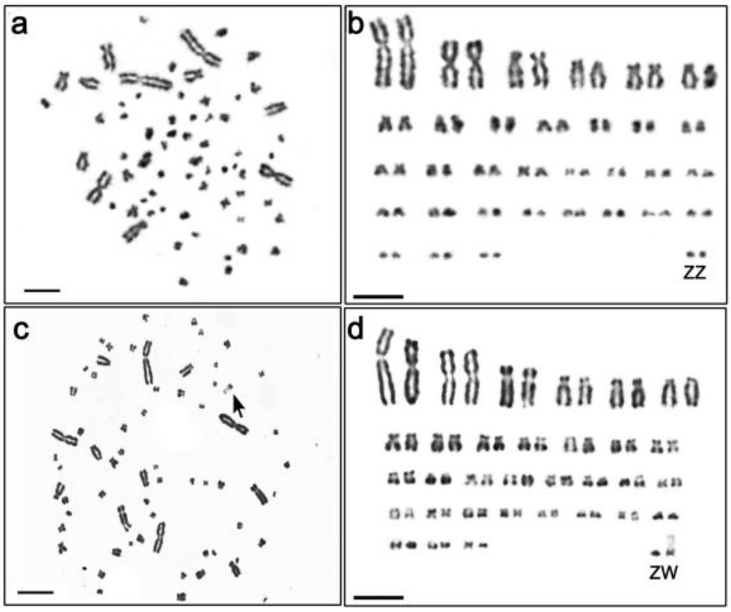

Figure S5. ZZ/ZW-type micro-ex chromosomes in Pelodiscus sinensis. (a, b) Giemsa-stained male metaphase karyotypes. (c, d) Giemsa-stained female metaphase karyotypes. The arrow indicates a female-specific chromosome. Scale bars are 10 μm.

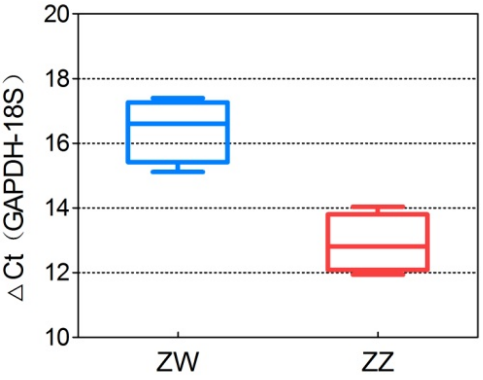

Figure S6. Quantification of the 18S rRNA repeats in ZW/ZZ *Pelodiscus sinensis*. Copy number of the 18S rRNA was normalized against GAPDH using the comparative Ct method of normalization (Ratio (18S/GAPDH) = $2^{\text{Ct GAPDH - Ct 18S}}$). A higher value of ΔCt (GAPDH - 18S) indicates a larger number of copies of 18S rRNA repeats.
